# SUMOylation Is Associated with Aggressive Behavior in Chondrosarcoma of Bone

**DOI:** 10.3390/cancers13153823

**Published:** 2021-07-29

**Authors:** Jessie S. Kroonen, Alwine B. Kruisselbrink, Inge H. Briaire-de Bruijn, Olaejirinde O. Olaofe, Judith V. M. G. Bovée, Alfred C. O. Vertegaal

**Affiliations:** 1 Department of Cell and Chemical Biology, Leiden University Medical Center, 2333 ZA Leiden, The Netherlands; J.S.Kroonen@lumc.nl; 2 Department of Pathology, Leiden University Medical Center, 2333 ZA Leiden, The Netherlands; A.B.Kruisselbrink@lumc.nl (A.B.K.); I.H.Briaire-de_Bruijn@lumc.nl (I.H.B.-d.B.); oolaofe@oauife.edu.ng (O.O.O.)

**Keywords:** SUMO, chondrosarcoma, cell cycle, ML792, survival

## Abstract

**Simple Summary:**

SUMO is a ubiquitin-like post-translational modification important for many cellular processes and is suggested to play a role in cancer cell cycle progression. The aim of our study is to understand the role of SUMOylation in tumor progression and aggressiveness. Chondrosarcoma of bone was employed as a model to investigate if SUMOylation contributes to its aggressiveness. We confirmed that SUMO expression levels correlate with aggressiveness of chondrosarcoma and disease outcome. Inhibition of SUMOylation showed promising effects on reduction of chondrosarcoma growth in vitro. Our study implies that SUMO expression could be used as a potential biomarker for disease outcome in chondrosarcoma.

**Abstract:**

Multiple components of the SUMOylation machinery are deregulated in various cancers and could represent potential therapeutic targets. Understanding the role of SUMOylation in tumor progression and aggressiveness would increase our insight in the role of SUMO in cancer and clarify its potential as a therapeutic target. Here we investigate SUMO in relation to conventional chondrosarcomas, which are malignant cartilage forming tumors of the bone. Aggressiveness of chondrosarcoma increases with increasing histological grade, and a multistep progression model is assumed. High-grade chondrosarcomas have acquired an increased number of genetic alterations. Using immunohistochemistry on tissue microarrays (TMA) containing 137 chondrosarcomas, we showed that higher expression of SUMO1 and SUMO2/3 correlates with increased histological grade. In addition, high SUMO2/3 expression was associated with decreased overall survival chances (*p* = 0. 0312) in chondrosarcoma patients as determined by log-rank analysis and Cox regression. Various chondrosarcoma cell lines (*n* = 7), especially those derived from dedifferentiated chondrosarcoma, were sensitive to SUMO inhibition in vitro. Mechanistically, we found that SUMO E1 inhibition interferes with cell division and as a consequence DNA bridges are frequently formed between daughter cells. In conclusion, SUMO expression could potentially serve as a prognostic biomarker.

## 1. Introduction

**Figure 1:**
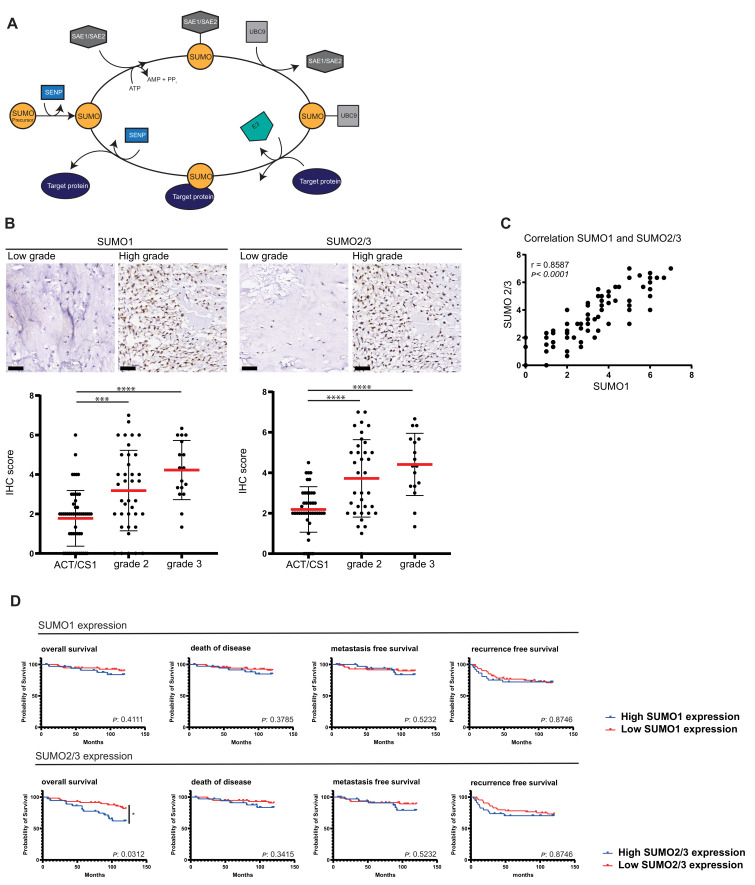
SUMO expression levels correlate with increasing histological grade of chondrosarcoma tumors and, thus, tumor aggressiveness, and poorer disease outcome. (**A**) Cartoon of the SUMOylation cycle. The SUMO precursor proteins are cleaved by SENPs to produce mature SUMO proteins. Subsequent SUMOylation of a target occurs via an enzymatic cascade involving an E1 (activating), E2 (conjugating), and E3 (ligase) enzyme. (**B**) Expression levels of SUMO1 and SUMO2/3 in chondrosarcoma primary tumors; atypical cartilage tumor/chondrosarcoma grade 1 (ACT/CS1) and grade 2 and grade 3 (high grade). Scale bars represent 50 µm. Dot-plots represent IHC scoring for SUMO1 and SUMO2/3 on tissue microarrays presented as mean with standard deviation. ***: *p* < 0.001, ****: *p* < 0.0001. Each dot represents the average of three cores per tumor. *p* values were calculated using one-way ANOVA followed by Tukey’s multiple comparison. (**C**) The correlation between SUMO1 and SUMO2/3 levels is shown for every tumor sample separately. Correlation calculation of SUMO1 vs. SUMO2/3 results in an R of 0.8584 and *p* < 0.0001. (**D**) Survival curves showing overall survival, death of disease, metastasis free survival and recurrence free survival, displaying high expression of SUMO1 or SUMO2/3 versus low expression of SUMO1 or SUMO2/3. SUMO expression was determined based on the scores of the TMAs from **B.** Log-rank (Wilson–Cox) analysis was used to calculate significance. *: *p* < 0.05 Cox regression analysis for the survival data shown in **D** is depicted in Table 1.

Post-translational modifications (PTMs) regulate virtually all cellular processes. PTMs include small chemical modifications, such as phosphorylation and modifications by small proteins belonging to the ubiquitin family, including SUMO (Small Ubiquitin-like modifier) [1,2]. SUMO can be covalently attached to target proteins and alter their activity, localization, interactions with other proteins, and/or half-life. SUMOylation is the enzymatic cascade that enables conjugation of SUMO to a target protein via an activating enzyme (E1; SAE1/UBA2), a conjugating enzyme (E2; UBC9 also known as UBE2I) and a ligase (E3). SUMOylation is reversible; SUMOs can be removed from substrates by SUMO specific proteases (SENPs) (Figure 1A). Mammals express at least three different SUMO family members, SUMO1–3. SUMO2 is the most abundant SUMO family member and is essential for embryonic development [3]. SUMO is predominantly regulating nuclear processes, including protein trafficking [4], gene expression, genome stability [5,6], and cell cycle control. SUMO signaling has been implicated in cancer. Many different types of cancer show deregulation of one or more components of the SUMO machinery, which predominantly results in an increase in SUMOylation [7,8].

Mice deficient for the SUMO conjugating enzyme UBC9 die at the early post-implantation stage due to major chromosome condensation and segregation defects [9]. Cell cycle progression defects were also observed in mutants of UBC9, SUMO E3 ligase GEI-17, and SUMO protease ULP-4, leading to severe defects, including embryonic arrest in *C. elegans*, potentially caused by chromosome segregation defects [10]. In addition, it was shown that SUMO specific proteases are also essential for proper cell cycle progression [11]. Knockdown of SUMO specific proteases SENP5 or SENP6 caused inhibition of cell proliferation. Interestingly, SENP6 regulates kinetochore assembly and consequently proper chromosomal segregation [12,13,14]. Combined, these studies define major roles for the SUMOylation pathway in cell cycle progression. 

Mechanistically, a number of SUMO targets have been identified as key substrates that mediate the roles of SUMOylation in the cell cycle [15]. FOXM1, essential for G2/M cell cycle phase transition and mitotic progression is a SUMO target and requires SUMOylation for full transcriptional activity [16]. Contrary to this activating role of SUMOylation on FOXM1, it was also proposed that SUMOylation of FOXM1 can inhibit its activity via the anaphase promoting complex cyclosome with Cdh1 as co-factor (APC/C-Cdh1) mediated ubiquitination and degradation, consequently causing mitotic delay [17]. Another SUMO target involved in cell cycle progression is DNA Topoisomerase IIα (TopoIIα). TopoIIα is a major regulator of faithful chromosome segregation, through resolving entangled DNA and via controlling checkpoint activation during cell cycle progression. SUMOylation of TopoIIα is needed for proper decatenation of centromeric DNA. SUMOylated TopoIIα regulates the localization of the Chromosomal Passenger Complex (CPC) via recruiting Haspin at the mitotic centromeres. Localization of the CPC to the mitotic centromeres is crucial for error-free chromosome segregation [18,19,20].

Furthermore, crosstalk of SUMO with other PTMs is important for progression through the cell cycle [21]. The regulation of the ubiquitin E3 ligase APC/C by SUMOylation is an intriguing example of crosstalk between SUMO and ubiquitin. SUMO can regulate the ubiquitination activity of the APC/C, which is critical for metaphase to anaphase transition during cell cycle progression [22,23]. SUMO modification of APC4 structurally alters the APC/C, reducing inhibition by the mitotic checkpoint complex [24]. 

These examples illustrate SUMOs roles in cell cycle progression and suggest potential for SUMOylation inhibition to block cancer cell proliferation. 

Conventional chondrosarcomas are a group of malignant bone tumors in which the tumor cells form cartilage. Most chondrosarcomas are located in the medulla of the bone called central chondrosarcoma, a small subset of which arises secondary to benign enchondromas. Less often, chondrosarcomas arise at the surface of bone (peripheral chondrosarcoma) within the cartilaginous cap of benign osteochondromas [25]. Histological grading is used to predict biological behavior and outcome. Atypical cartilaginous tumor/chondrosarcoma grade I (ACT/CS1) has a 10-year survival of 88%, while grade II chondrosarcomas have a 10-year survival of 62%, and grade III chondrosarcomas have a 10-year survival of only 26% [26]. A subset of tumors (~10%) contains areas of dedifferentiation, which is associated with a very poor disease outcome. Thus, aggressiveness increases with increasing histological grade. A multistep progression model is assumed, in which tumors acquire an increased number of genetic alterations upon progression towards higher histological grade [27]. Isocitrate dehydrogenase (IDH1/2) mutations were found in 50% of central chondrosarcomas and are considered an early event [28,29,30]. IDH1/2 play a role in the Krebs cycle, and mutations lead to an accumulation of the oncometabolite D-2-hydroxyglutarate (D2-HG), which causes epigenetic changes and metabolic alterations [31,32]. In high-grade chondrosarcoma, the p53 and Rb pathways are deregulated and complex genomic alterations are frequently found, which includes, amongst many others, amplification of MYC [33]. 

Here we aimed to understand the role of SUMOylation in tumor progression and aggressiveness. We employed chondrosarcoma as a model to investigate if SUMOylation contributes to its aggressiveness. A single specific SUMO E1 inhibitor was used to investigate the druggability of the SUMO pathway in this model [34]. 

## 2. Material & Methods

### 2.1. Compound

ML792 [34] was obtained from Millennium Pharmaceuticals, Inc. (Cambridge, MA, USA), a wholly owned subsidiary of Takeda Pharmaceutical Company Limited (Tokio, Japan), and was dissolved in DMSO for in vitro usage. 

### 2.2. Tissue Microarray (TMA) and Clinicopathological Data

Previously constructed tissue micro arrays (TMAs) containing 137 conventional chondrosarcomas (92 central of which 42 ACT/grade I, 36 grade II, 14 grade III, and 45 peripheral including 31 ACT/grade I, 11 grade II, 3 grade III) [35] were stained for SUMO1 (4930P, Cell Signaling Technology, Leiden, The Netherlands) and SUMO2/3 (2277 rabbit, Eurogentec, Seraing, Belgium) [36]. Details on the construction of the TMA and clinicopathological data for this patient series were reported previously [35]. A waiver of consent was obtained from the medical ethical evaluation committee (protocol number: B17.020 v2). All specimens in this study were handled according to the ethical guidelines described in ‘Code for Proper Secondary Use of Human Tissue in The Netherlands’ of the Dutch Federation of Medical Scientific Societies. 

### 2.3. Immunohistochemistry

Immunohistochemistry was performed on the TMAs. TMAs were deparaffinized and rehydrated and endogenous peroxidase was blocked. Antigen retrieval was performed with citrate pH 6.0 and treated for 10 min in the microwave. Subsequently the slides were stained with anti-SUMO2/3 (2277 rabbit, Eurogentec, 1:16,000). Poly-HRP-GAM/R IgG (DVPO110HRP, ImmunoLogic, Duiven, The Netherlands) was used as secondary antibody and liquid DAB+ substrate Chromogen System (K3468, DAKO; Carpinteria, CA, USA) was used for visualization. Slides were counter stained with haematoxylin [37]. 

Slides were scored independently by two pathologists (JVMGB and RO). The immunohistochemistry score was calculated as the sum of staining intensity (0–3, no staining—strong intensity) and percentage of positive tumor cells (0–4, 0–(75–100)%) as published previously [35]. Discrepancies in scoring were re-evaluated by both investigators together to reach consensus.

### 2.4. Analysis of Chondrosarcoma TMA Data Set 

To conduct survival analysis, patient data from the TMA data set was dichotomized into high and low SUMO1 or SUMO2/3 expression groups, according to the immunohistochemistry scoring. The median was chosen as the cut-off. Differences in overall survival, disease related survival, metastasis-free survival, and recurrence-free survival were estimated using Kaplan–Meier curves and log-rank analysis using the Prism 6 GraphPad software version 9.0.1, San Diego, CA, USA). Univariate and multivariate Cox regression analysis was used to investigate independent variable grading using IBM SPSS statistics 25. Grading was used as categorical variable. Analysis displayed hazard ratio (B) for SUMO expression, hazard ratio (B) for grading in which risk of ACT/CS1 and 2 was compared to grade 3, and SUMO expression hazard per grade. *p-*value significance corresponds to *p* < 0.05. 

### 2.5. Analysis of Chondrosarcoma mRNA Expression Data Set 

Survival analysis on the mRNA expression from chondrosarcoma tumor sample data set containing 102 patient samples from Nicolle et al. 2019 [38] were conducted in samples of which clinical outcome was reported (*n* = 90), containing 8 benign, 16 dedifferentiated, 17 grade 1, 39 grade 2, and 17 grade 3 patient samples. mRNA gene expression for SUMO1, SUMO2, SAE1, UBA2, and UBE2I was dichotomized into high and low expression groups using the median of the data as cut-off. Differences in overall survival were calculated using Kaplan–Meier curves and log-rank analysis in Prism 6 GraphPad software. Univariate Cox regression analysis was used to investigate hazard for gene expression displayed as dichotomized data-set as used in the Kaplan–Meier analysis and gene expression as a continuous value calculating hazard according to increasing values per sample. IBM SPSS statistics 25 was used to conduct Cox regression analysis. *p-*value significance corresponds to *p* < 0.05. 

### 2.6. Analysis of Soft Tissue Sarcoma Data from the TCGA Repository

The TCGA-SARC data set from The Cancer Genome Atlas (TCGA, National Cancer Institute, Bethesda, MD, USA) repository contains a heterogeneous group of soft tissue sarcoma samples (*n* = 259). Correlations between mRNA expression and survival were calculated using Cox proportional hazard regression and to draw Kaplan–Meier plots. Cut-offs were computed as best-cut offs out of the data set by the kmplot.com algorithm [39]. *p-*value significance corresponds to *p* < 0.05. 

### 2.7. Cell Culture

A panel of chondrosarcoma cell lines was used, including CH2879 obtained from Professor A Llombart-Bosch (University of Valencia, Valencia, Spain) [29,40], SW1353 obtained from ATCC [29], L2975 [29,41], JJ012 obtained from Dr. JA Block (Rush University Medical Centre, Chicago, IL, USA) [29,42], HT1080 [30,43], and NDCS1 obtained from Dr. T Ariizumi (Niigata University Graduate School of Medical and Dental Sciences, Niigata, Japan) [29,44]. Cells were cultured in RPMI 1640 medium (Life Technologies) with 10% Fetal Bovine Serum (FBS, South America Origin, Biowest, Amsterdam, The Netherlands) and 5% Penicillin–Streptomycin (P/S, Life Technologies, Carlsbad, CA, USA). CH3573 was obtained from Professor A Llombart Bosch (University of Valencia, Valencia, Spain) [45] and was cultured in RPMI 1640 medium with 20% FBS and 5% P/S. The hTERT RPE-1 (retina pigment epithelial) cell line was purchased from ATCC, VH10 cells (human foreskin fibroblasts) were a kind gift from AG Jochemsen (Leiden University Medical Center, Leiden, The Netherlands). Cells were cultured in a humidified incubator at 37 °C and 5% CO_2_. Mycoplasma tests were performed on a regular basis and cell identities were confirmed using STR profiling (GenePrint 10 System, Promega, Madison, WI, USA).

### 2.8. Cell Viability Assay

Chondrosarcoma cells were seeded in a 96-well plate (1500–5000/well). After overnight adherence, cells were treated with increasing concentrations (0, 50, 150, 250, 500, 1000 nM) of ML792 for 72 h using DMSO as control. To correct for differences in doubling times of the different cell lines, three extra wells were seeded per cell line at day 0 and measured at the start of the treatment, correcting for the growth difference between seeding and start of treatment. [46] Presto blue viability reagent (A13261, Thermo Fisher Scientific; (Waltham, MA, USA) was added to the cells in a 1:10 dilution in culture medium, old medium was removed. After 60 minutes of incubation at 37 °C and 5% CO_2_ fluorescence was measured using a plate reader (Victor X3, Perkin Elmer, Waltham, MA, USA) at 544/591 nm. All experiments were independently performed three times, using three technical triplicates per sample. Cell count is assumed to be following a similar pattern and not differentially affected and was therefore not evaluated [47].

### 2.9. Colony Formation Assay

Cells were seeded at a low density (3000–6000 cells/well) in a 6-well plate. After overnight adherence, cells were treated once or on day 1, 4, 7, and 10 after seeding with the indicated concentrations (0, 50, 150, 250, 500, 1000 nM) of ML792 using DMSO as control. Colonies were grown for 10 to 14 days, medium was removed from the wells, and wells were washed with PBS. Cells were fixed with ice-cold methanol for 20 minutes at −20 °C and stained with crystal violet (0.05 mg/mL) for 30 minutes. Excess crystal violet was removed, plates were washed with water and left to dry overnight. Quantification of crystal violet staining was carried out by dissolving crystal violet retained by the colonies in methanol for 30 minutes. Absorbance of crystal violet was measured at 595 nm on a plate reader (Victor X3, Perkin Elmer).

### 2.10. Western Blot

Conjugation of SUMO2/3 (1:500, mouse monoclonal AB_2198421, University of Iowa, Iowa City, IA, USA) and ubiquitin (1:1000, mouse sc8017, Santa Cruz, Dallas, TX, USA) and expression of UBA2 (1:1000, rabbit monoclonal, D15C11, Cell Signaling Technology), UBC9 (1:1000, mouse, 610748, BD Biosciences, San Jose, CA, USA), c-MYC (1:1000, rabbit monoclonal, Y69, Abcam, Cambridge, Unicted Kingkom), and _Ƴ_-tubulin (1:1000, mouse monoclonal, clone GTU-88, T6557, Sigma, Saint Louis, MO, USA) were determined by immunoblotting of lysates from cells of our chondrosarcoma panel (see cell culture). Lysates were prepared using SNTBS buffer (2% SDS, 1% NP40, 50 mM Tris pH 7.5, 150 mM NaCl) and boiled at 100 °C for 10 minutes, before snap freezing. Proteins were separated on 4–12% Bolt™ Bis–Tris gradient gels (Thermo Fisher Scientific, Waltham, MA, USA). Proteins were transferred to Amersham Protran Premium nitrocellulose membranes (Sigma Aldrich). Ponceau S staining was performed to confirm equal loading of samples. Membranes were blocked in PBS-T (0.05% Tween, Merck Millipore, Burlington, MA, USA) containing 5% milk powder for 60 minutes. Primary antibodies were diluted in PBS-T (0.05% Tween) and incubated with the membrane at 4 °C overnight. Donkey anti-rabbit IgG-HRP and goat anti-mouse IgG-HRP secondary antibodies were diluted 1:2500 in PBS-T (0.05% Tween) containing 5% milk and detected using chemo luminescence with Pierce ECL Plus Western Blotting substrate (catalog number. 32132, Thermo Fisher Scientific). 

### 2.11. Cell Cycle Analysis by Flow Cytometry

CH2879, JJ012, and NDCS1 cells were seeded in 10 cm dishes (0.5–1 × 10^6^ cells/dish). Cells were allowed to adhere overnight. The following day cells were treated with 1 µM ML792 for 24 h. Cells were harvested and washed with ice cold PBS, next cells were fixed in 70% ethanol at −20 °C overnight. Cells were stained with propidium iodide in PBS/1% Bovine serum albumin (BSA)/0.05% Tween at 4 degrees Celsius overnight. Analysis was performed with BD LSRII in BD FACSDiva software (BD biosciences, San Jose, CA, USA). Within each analysis we measured 10,000 events. Data were processed with FlowJow_V10.7.1, visualizing distribution of DNA content. 

### 2.12. Microscopy

CH2879 cells (a-synchronous) were seeded on glass coverslips at 50,000 cells per well in a 6-well plate containing one glass coverslip. Following overnight adherence, cells were treated for 24 and 48 hours with 250, 500, and 1000 nM of ML792 or with a DMSO 0.1% control. Cells were fixed on the coverslips with 4% PFA for 20 minutes, followed by 5 PBS washes. Samples were permeabilized using 0.1% Triton for 15 minutes and washed two times with PBS and two times with PBS-T (0.05% Tween). Next samples were blocked using TNB buffer (0.1 M Tris-HCl, pH 7.5, 0.15 M NaCl, 0.5% (w/v) blocking reagent (PerkinElmer FP1020, Waltham, MA, USA). Samples were dehydrated using 70, 90, and 100% ethanol followed by mounting of the slides with ProLong™ Gold Antifade Mountant (Fisher Scientific) with DAPI. Cells were imaged using an upright microscope DM6B (Leica Microsystems, Wetzlar, Germany). Three replicates were performed for each condition; for each replicate 15 fields with 20 to 25 cells per field on average were imaged. CH2879, NDCS1, and JJ012 cells were treated with DMSO or 1000 nM ML792 for 24 h, processed as described above, stained with Hoechst and imaged on SP8 confocal microscopy (Leica Microsystems). 

### 2.13. Statistical Analysis

For the difference in IHC scores of TMAs upon SUMO expression and for the difference in mRNA expression, a *p* value was calculated using one-way ANOVA followed by Tukey’s multiple comparison using the Prism 6 GraphPad software. 

Dose response curves and IC_50_ values were determined using Prism 6 GraphPad software (Figure 3B,D). Differences between samples (Figure 3E) were calculated using two-tailed t-tests, also employing Prism 6 GraphPad software. Differences between protein expression levels of cell lines (Figure 4D) were calculated using one-way ANOVA followed by Tukey’s multiple comparison, using Prism 6 GraphPad software.

## 3. Results

### 3.1. High Expression Levels of SUMO1–3 Correlate with Aggressiveness in Chondrosarcoma

In order to understand the relation between SUMO expression and tumor aggressiveness, the expression levels of different SUMO family members were evaluated in a series of primary tumor tissue of chondrosarcoma patients. Tissue microarrays (TMAs) were used containing 137 tumors of various histological grades and stained with antibodies against SUMO1 or SUMO2/3. Chondrosarcomas grade 2 and 3 expressed significantly more SUMO compared to ACT/CS1 (SUMO1 expression: ACT/CS1 vs. grade 2 *p* = 0.0004, ACT/CS1 vs. grade 3 *p* < 0.0001, SUMO2/3 expression: ACT/CS1 vs. grade 2 *p* < 0.0001, ACT/CS1 vs. grade 3 *p* < 0.0001) (Figure 1B). SUMO1 and SUMO2/3 were similarly expressed within samples (Figure 1C). Furthermore, a similar difference in expression was shown for SUMOylation pathway components SAE1, UBA2, and UBC9 between low and high grade chondrosarcoma, upon analyzing mRNA expression data published in Nicolle et al. 2019 [38] (Figure S2A). Expression in the aggressive subset of dedifferentiated chondrosarcoma was separately visualized and showed also increased expression of SUMOylation pathway components comparable to grade 2 and 3 chondrosarcoma. We conclude that SUMO expression levels correlate with increasing histological grade of chondrosarcoma and thus aggressiveness.

### 3.2. High SUMO Expression Correlates with Poorer Survival

The chondrosarcoma TMAs in combination with clinical outcome data were used to investigate the relation between SUMO expression and disease outcome in chondrosarcoma patients (Figure 1D, S1). Increased SUMO2/3 expression levels significantly decreased overall survival chances (log-rank *p* = 0.0312, Cox regression 0.026), a similar trend is shown for SUMO1. Furthermore, a similar pattern is shown for risk of death of disease, metastases and recurrence. (Figure 1D, Table 1). Analyzing different tumor subsets, central chondrosarcoma and peripheral chondrosarcoma, separately showed that high SUMO2/3 expression significantly decreased overall survival in peripheral chondrosarcoma (*p* = 0.0298) (Figure S1). To investigate correlation of SUMO expression with grading and clinical outcome, we performed a multivariable Cox regression analysis. SUMO expression decreased risk within the grade subsets for patients dying of the disease and recurrence of the disease significantly (Table 1).

Our results stimulated us to investigate another set of chondrosarcoma samples published by Nicolle et al. 2019 [38]. This data set contains mRNA expression analysis of chondrosarcoma tumor samples (*n* = 102), and provides information on tumor histology and clinical outcome for 90 patients. We found a significant risk for poorer disease outcome with over expression of SUMO2 (log-rank *p* = 0.0569, Cox regression *p* = 0.003), both SUMO E1 enzyme subunits (SAE1 log-rank *p* = 0.0025, Cox regression *p* = 0.000, UBA2 log-rank *p* = 0.0002, Cox regression *p* = 0.002) and SUMO E2 enzyme (log-rank *p* = 0.0073, Cox regression 0.000) (Figure S2B and Table S1). A similar pattern of results was found upon analyzing a heterogeneous soft tissue sarcoma RNAseq and survival data set (*n* = 259) from the TCGA repository. A significant risk for poorer disease outcome was linked to overexpression of SUMO2, SUMO3 and both SUMO E1 subunits (Figure S3). We conclude that increased levels of SUMO pathway components are associated with poorer disease outcome in chondrosarcoma patients and can potentially be extended to more sarcoma subtypes.

### 3.3. SUMO E1 Inhibition Decreases SUMO-Conjugation in Chondrosarcoma Cell Lines

**Figure 2:**
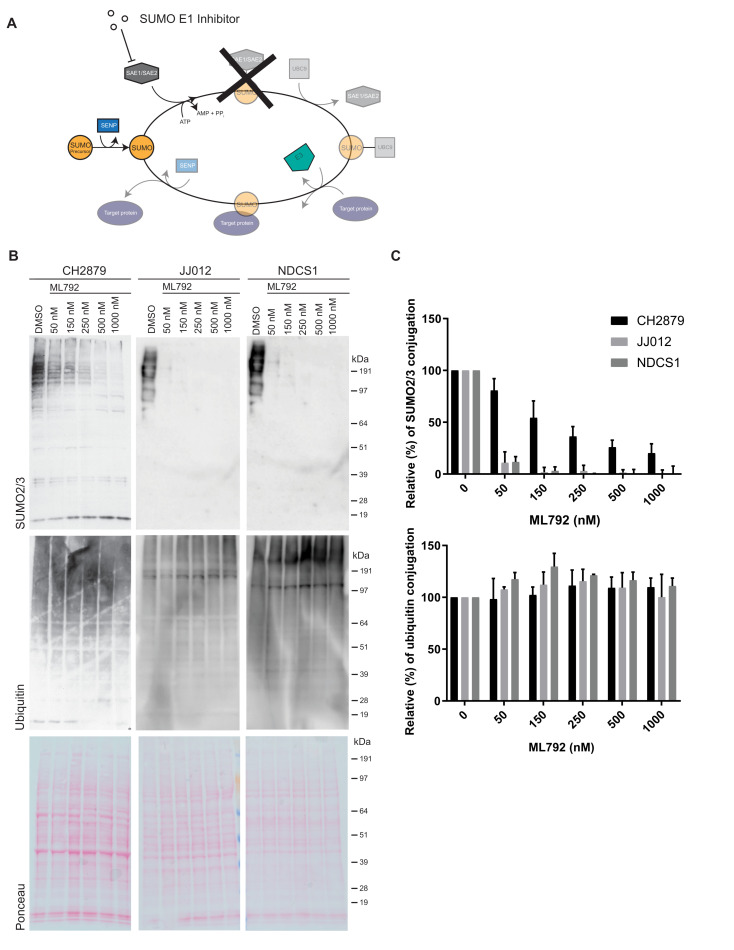
The SUMO E1 inhibitor ML792 blocks SUMO conjugation. (**A**) Cartoon of the function of the SUMO E1 inhibitor ML792. (**B**) Western blot analysis of SUMO2/3 and ubiquitin levels of the chondrosarcoma cell lines CH2879, JJ012, and NDCS1 treated with the indicated concentrations of the SUMO E1 inhibitor ML792 for 4 h compared to solvent control (DMSO 0.1%). Ponceau S staining was used as loading control. (**C**) Quantitative analysis of SUMO2/3 and ubiquitin conjugation of the corresponding western blots shown in B. Data represent mean with standard deviation (*n* = 3).

Subsequently, we investigated whether SUMOylation is important for the proliferation of chondrosarcoma cells in vitro. For this purpose, we employed the SUMO E1 inhibitor ML792 [34] (Figure 2A). ML792 forms a covalent adduct with SUMO via its C-terminus. The SUMO-ML792 adduct directly binds to and blocks the SUMO E1 enzyme to prevent SUMO conjugation [48]. We verified that ML792 inhibits SUMO conjugation in a dose-dependent manner in three different chondrosarcoma cell lines, CH2879, JJ012, and NDCS1 (Figure 2B,C). ML792 did not affect the related post-translational modification ubiquitination, demonstrating its specificity.

### 3.4. SUMO E1 Inhibition Reduces Cell Proliferation and Viability In Vitro

**Figure 3:**
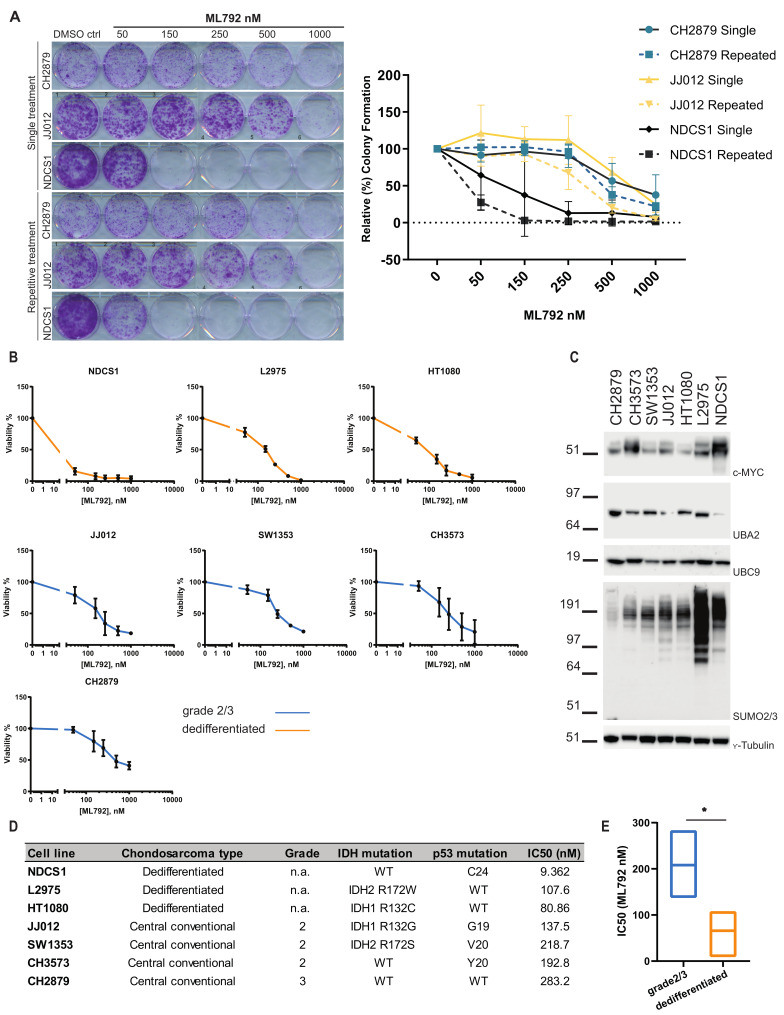
Inhibition of SUMO E1 via ML792 results in decreased proliferative capacity and cell viability. (**A**) Two-dimensional (2D) colony-formation assay (crystal violet). CH2879, JJ012, and NDCS1 cells were treated with ML792 cells using single treatment or repetitive treatment on day 1, 4, 7, and 10 after seeding. Colony-formation was quantified via measuring crystal violet staining. Data represent mean with standard deviation (*n* = 3). (**B**) Viability assay using PrestoBlue. Chondrosarcoma cell lines were treated with ML792 and incubated for 3 days. Relative cell viability is represented as mean with standard deviation (*n* = 3). (**C**) Expression levels of c-MYC, UBA2, UBC9 and conjugation of SUMO2/3 in lysates of untreated chondrosarcoma cell lines from Figure 3C. _Ƴ_-tubulin was used as loading control. Single representative images are shown (*n* = 3). Whole blots including intensity readings can be found in Figure S5 (**D**) Table displaying specifics of the cell lines from **B** including IC50 (nM) values. (**E**) Boxplots to compare IC50 values of dedifferentiated (*n* = 3) and grade 2/3 (*n* = 4) chondrosarcoma cell lines. Significance was calculated with a two-tailed *t*-test *: *p* < 0.05.

Next, we tested whether SUMO E1 inhibition blocked chondrosarcoma cell proliferation. For this purpose, we conducted colony formation assays. Cells were seeded at low density and treated with SUMO E1 inhibitor or DMSO to verify the effect of SUMO inhibition on cell proliferation. Inhibition of the SUMO E1 enzyme by ML792 led to a decrease in colony formation in all three tested chondrosarcoma cell lines, in a dose-dependent manner (Figure 3A). ML792 repetitive treatment was shown to be more effective over single treatment. Using the PrestoBlue cell viability assay, we confirmed that chondrosarcoma cell viability was affected by ML792, in a dose dependent manner, in a panel of several chondrosarcoma cell lines (*n* = 7). The NDCS1 cell line (derived from a dedifferentiated chondrosarcoma, *IDH* wild type) was most sensitive to SUMO inhibition with an IC_50_ of 9.4 nM ML792 and the CH2879 cell line (grade 3 chondrosarcoma, *IDH* wild type) was the least sensitive, IC_50_ 283 nM ML792 (Figure 3B,D). In addition, we found that non-malignant cells were less sensitive to ML792 treatment. RPE-1 cells had an IC_50_ of 377.5 nM ML792 and VH10 cells had an IC_50_ >1µM ML792 (Figure S4A). We conclude that SUMO E1 inhibition decreased chondrosarcoma cell proliferation and cell viability in a dose- and cell line dependent manner. Concerning subtype and mutational status of the cell lines in our panel, cell lines originating from dedifferentiated chondrosarcoma were most sensitive towards SUMO E1 inhibition (Figure 3B,D), IC50 values of dedifferentiated chondrosarcoma were significantly (*p* = 0.0221) lower compared to grade 2 or 3 chondrosarcoma cell lines (Figure 3E). No relation between sensitivity and *IDH* mutation or p53 mutation status was found (Figure S4B).

High expression of c-MYC in B-cell lymphoma was previously shown to correlate with sensitivity towards SUMO E1 inhibition [49]; therefore, we studied c-MYC expression in our panel of chondrosarcoma cell lines. NDCS1 and CH3573 expressed significantly higher c-MYC levels compared to CH2879, SW1353, JJ012, and HT1080 (Figure 3C, Figure S4C and S5). The NDCS1 cell line is most sensitive towards ML792 and the CH3573 cell line is among the least sensitive cell lines in our panel, indicating a lack of correlation between c-MYC levels and sensitivity towards SUMO E1 inhibition in chondrosarcoma. Expression levels of UBC9 and UBA2 were similar between the cell lines in our panel, whereas SUMO conjugation is consistently high in two of the most sensitive cell lines, NDCS1 and L2975, indicating that SUMO conjugation levels could potentially be used as biomarker to predict ML792 sensitivity.

**Figure 4:**
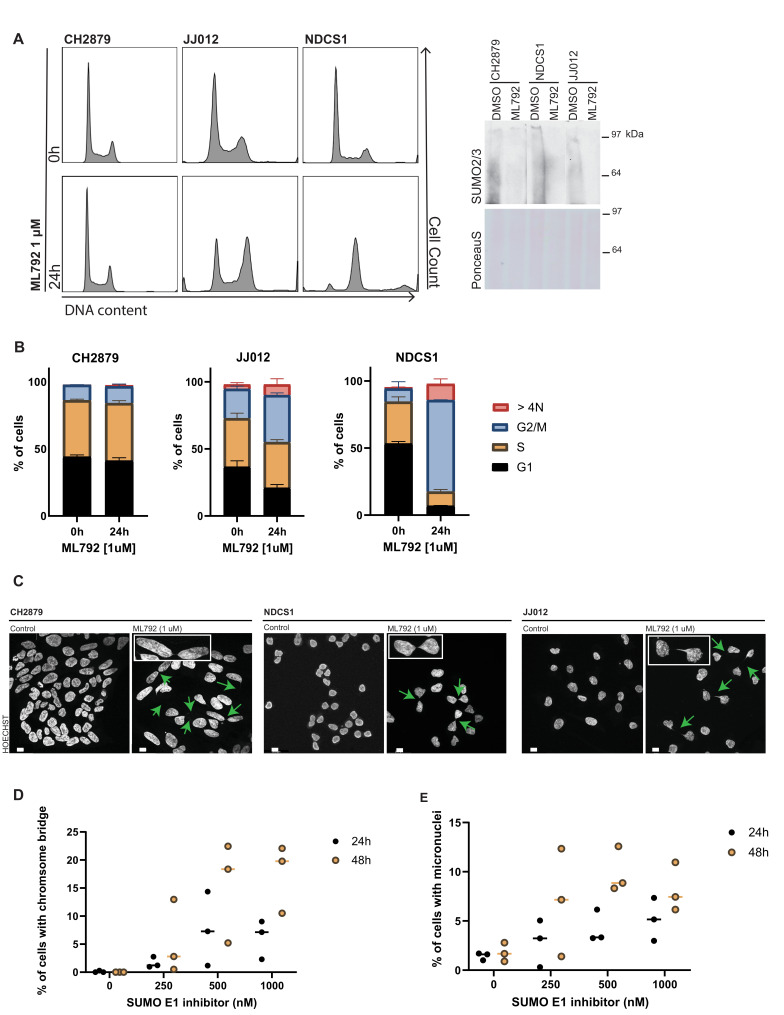
SUMO E1 inhibition leads to G2/M arrest and chromosome bridge formation. (**A**) Cell cycle analysis by flow cytometry of chondrosarcoma cell lines treated with ML792 for 24 h. Western blot analysis confirmed the inhibition of SUMO conjugation by the SUMO E1 inhibitor ML792. (**B**) Data representation of **A** in bar-graphs indicating G1, S, and G2/M pool of the cell lines for 0 or 24 hours of treatment as mean with standard deviation (*n* = 3). (**C**) Immunofluorescence images of a-synchronous CH2879, NDCS1, and JJ012 cells treated with ML792 for 24 h. Cells were stained with Hoechst to visualize DNA. Green arrows indicate the location of chromosome bridges. Insert: magnification of a chromosome bridge. Scale bars represent 10 µm. (**D**) Quantification of chromosome bridges in CH2879 cells upon treatment with the indicated concentration of the SUMO E1 inhibitor (ML792) for 24 and 48 h. For each replicate 15 images (approximately 200 cells total) per condition were analyzed. Data represent mean with standard deviation (*n* = 3). (**E**) Quantification of micronuclei present in CH2879 cells upon treatment with the indicated concentration of the SUMO E1 inhibitor (ML792) for 24 and 48 h. For each replicate 15 images (approximately 200 cells total) per condition were analyzed. Data represents mean with standard deviation (*n* = 3).

To investigate the effect of ML792 on cell proliferation in more detail, we performed DNA content analysis using flow cytometry. NDCS1 and JJ012 cells showed a striking increase in the G2/M pool of cells after 24 hours of ML792 treatment and a little increase in the G2/M pool upon ML792 treatment was noted for CH2879 (Figure 4A,B). Furthermore, an increase was also shown for cells with a more than 4N DNA content. Subsequently, microscopy analysis revealed that chromosome bridges between daughter cells and micronuclei were formed upon SUMO E1 inhibition in CH2879, NDCS1, and JJ-012 cells (Figure 4C). Increasing concentrations of ML792 correlated with an increase in chromosome bridges and micronuclei in CH2879 cells over 24- and 48-hour periods (Figure 4D,E). Our results shown here are consistent with results obtained with ML792 in other types of tumor cell lines [34].

## 4. Discussion

We investigated the contribution of SUMO expression to tumor progression, aggressiveness, and relation to clinical outcome in chondrosarcomas. We demonstrated increased expression of SUMO1 and SUMO2/3 in high-grade chondrosarcomas, associating SUMO expression with tumor progression and increased aggressiveness. Subsequently, we showed that high SUMO2/3 expression correlates with poorer survival. In addition, using an mRNA expression dataset containing chondrosarcoma tissue samples and the TCGA mRNA expression data set containing a heterogeneous set of soft tissue sarcomas, focusing on SUMOylation cascade components, showed comparable results, correlating high SUMO2/3 protein and high SUMO E1 and high E2 enzyme expression to poorer disease outcome. Strikingly SUMO1 expression did not correlate with poorer disease outcome in all datasets. Combined, the data show that high SUMO cascade components potentially predict poorer disease outcome in chondrosarcoma patients and even in a more diverse set of sarcoma patients, indicating that SUMO pathway expression levels may serve as a prognostic biomarker. 

In vitro, a panel of chondrosarcoma cell lines were found to be sensitive to the SUMO E1 inhibitor ML792 and dedifferentiated chondrosarcoma cell lines were particularly sensitive to loss of SUMO conjugation. Increasing the number of cell lines of the panel would strengthen this claim in future research. SUMO E1 inhibition resulted in an increase in G2/M cells and > 4N cells, frequent occurrence of chromosome bridges between daughter cells, and a reduction in chondrosarcoma cell viability and reduced colony formation [23,34,50]. The block in chondrosarcoma cell proliferation and increase of G2/M cells and >4N cells in response to SUMO E1 inhibition that we observed in vitro can potentially be explained by the formation of DNA bridges between daughter cells. The formation of these bridges is expected to result in damaged DNA and will ultimately prevent cell cycle progression. Critical roles for SUMO to maintain chromosome integrity have previously been found [51]. Interestingly, SUMOylation of TopoII is needed for proper centromeric cohesion. In yeast, SMT3/SUMO protease SMT4 prevents precocious sister chromatid separation via deSUMOylating TopoII and Pds5P. DeSUMOylation of TopoII and Pds5P is needed to prevent precocious sister chromatid separation [52,53]. As mentioned in the introduction, TopoIIα needs to be SUMOylated to recruit the CPC to the centromere and facilitate proper decatenation of the sister chromatids and start chromosome segregation [18,19,20]. Thus, correct timing of TopoII and Pds5P SUMOylation as well as deSUMOylation might be required for proper mitosis. DNA bridges observed upon SUMO E1 inhibition could be the result of decreased SUMO conjugation of TopoII and Pds5P resulting in decatenation defects. 

In addition, two SUMO E3 ligases, RanBP2 and MMS21, are linked to chromosome resolution [54,55]. Mutant mice with low levels of RanBP2 and knockdown of MMS21 show delayed cell cycle progression and aneuploidy. RanBP2 is responsible for the SUMOylation of TopoIIα, which is critical for centromeric cohesion as described above. Consequently, insufficient SUMOylation of TopoIIα via RanBP2 results in impaired chromosome segregation [54]. MMS21 dependent SUMOylation is suggested to be important for chromosome cohesion [56] and knockdown of MMS21 results in broken chromosomes [55]. Overall, decreased SUMO conjugation, by SUMO E1 inhibition or due to low SUMO E3 ligase activity, or increased SUMOylation due to the absence of a SUMO protease, leads to defects in cohesion and decatenation of sister chromatids. If sister chromatids are still connected and the cell continues through mitosis, this can result in DNA bridges between daughter cells as shown in our experiments.

Furthermore, we showed that dedifferentiated chondrosarcoma cell lines were particularly sensitive for SUMO E1 inhibition and tumor samples from dedifferentiated chondrosarcoma show increased expression of the SUMOylation cascade enzymes. As mentioned previously, metastatic and inoperable chondrosarcoma is difficult to treat since they are highly resistant towards conventional chemotherapy and radiotherapy. Dedifferentiated chondrosarcoma is the most aggressive subtype with approximately 18% overall survival rate after 5 years [57,58,59]. Several potential therapeutic targets in dedifferentiated chondrosarcoma have been identified, including IDH1/2 mutations in approximately half of the cases [60]. IDH1/2 mutations lead to an accumulation of the oncometabolite D-2-hydroxyglutarate (D2-HG) [31,32]. Targeting glutaminolysis, which generates α-ketoglutarate as part of the Krebs cycle, and the precursor for D-2-HG is an interesting therapeutic route [61]. One more target in the metabolic process recently explored in chondrosarcoma therapy is m-TOR, showing in vitro and in vivo efficacy of m-TOR inhibition [62,63]. m-TOR inhibition is currently in a phase II clinical trial for conventional, mesenchymal, and dedifferentiated chondrosarcomas (NCT02821507). 

Another therapeutic approach could be to reactivate apoptotic pathways via inhibiting anti-apoptotic proteins Bcl-2 and Bcl-xL, which supposedly play an important role in chondrosarcoma chemoresistance, because inhibition of anti-apoptotic proteins increased sensitivity towards cisplatin and doxorubicin [64]. Inhibition of one single target may not be sufficient as resistance mechanisms may develop, and multi-target/combination therapy is needed [65]. Therefore, it would be of interest to investigate potential combination treatments that include SUMOylation inhibition as potential novel therapeutic strategies for chondrosarcoma. Furthermore, our results suggest that the dedifferentiated subtype may be particularly sensitive to the SUMO E1 inhibitor. 

Several studies have shown that intervening with the SUMOylation pathway in different types of cancer leads to a decrease in cancer cell growth [50,66,67]. In addition to ML792, other small molecule SUMO E1 inhibitors COH000, ML93, and TAK981, an analogue of ML792, have shown to inhibit tumor growth in vitro and in vivo [68,69,70]. TAK981 has entered clinical trials in combination with pembrolizumab for metastatic solid tumors and in combination with rituximab for non-Hodgkin’s lymphoma (NCT03648372, NCT04074330, and NCT04381650). Broadening our perspective to other ubiquitin pathway inhibitors shows that the Neddylation E1 inhibitor MLN4924 (Pevonedistat) is already in phase III clinical trials (NCT03268954, NCT04090736) and results have been published for phase I clinical studies [71,72]. In addition, inhibitors for the ubiquitin E1 enzyme also show promising anti-tumor effects in preclinical models [73]. Together these inhibitors highlight the increasing therapeutic potential to target tumors via blocking ubiquitin and ubiquitin-like post translational modifications. 

## 5. Conclusions

We found that SUMO2/3 expression correlates with high histological grade in chondrosarcoma and predicts poor clinical outcome. Therefore, SUMO2/3 expression could potentially serve as a biomarker for disease outcome. Since dedifferentiated chondrosarcoma cell lines are particularly sensitive towards SUMO E1 inhibition in vitro, future research could address if this highly aggressive chondrosarcoma subtype can be treated with SUMO E1 inhibitors in vivo. Furthermore, novel therapeutic combination strategies could be explored. From our work, we conclude that SUMO2/3 expression could potentially serve as a prognostic biomarker. It remains to be investigated if SUMO2/3 expression can also be used as a predictive biomarker for SUMO E1 inhibitor treatment. 

## Figures and Tables

**Table 1:** Univariate* and multivariate^#^ Cox regression analysis results of variates influencing survival.

Clinical Association	Variable	Hazard Ratio (B)	CI (95%) (exp(B))	*p* Value
**10-year survival**				
*SUMO1* expression *		0.312	0.614–0.3042	0.444
*ACT/CS1* (compared to grade 3) ^#^		−2.488	0.026–0.267	0.000
*Grade 2* (compared to grade 3) ^#^		−1.198	0.122–0.744	0.009
*SUMO2/3*exp (grade dependent) ^#^		−0.538	0.245–0.1392	0.225
*SUMO2/3* expression *		0.95	1.117–5.979	0.026
*ACT/CS1* (compared to grade 3) *^#^*		−2.692	0.014–0.338	0.001
*Grade 2* (compared to grade 3) *^#^*		−1.155	0.109–0.914	0.034
*SUMO1*exp (grade dependent) ^#^		−0.262	0.254–2.331	0.644
**10-year survival (death of disease)**				
*SUMO1* expression *		0.527	0.517–5.552	0.384
*ACT/CS1* (compared to grade 3) ^#^		−3.773	0.003–0.205	0.001
*Grade 2* (compared to grade 3) ^#^		−2.047	0.032–0.517	0.004
*SUMO2/3*exp (grade dependent) ^#^		−0.685	0.144–1.766	0.284
*SUMO2/3* expression *		0.892	0.687–8.654	0.168
*ACT/CS1* (compared to grade 3) *^#^*		−15.087	0.00–8.075E136	0.929
*Grade 2* (compared to grade 3) *^#^*		−3.075	0.007–0.319	0.002
*SUMO1*exp (grade dependent) ^#^		−1.933	0.23–0.896	0.038
**Metastasis-free survival (10-year)**				
*SUMO1* expression *		0.523	0.515–5.528	0.388
*ACT/CS1* (compared to grade 3) ^#^		10.145	0.00–1.112E+120	0.940
*Grade 2* (compared to grade 3) ^#^		10.991	0.00–2.586E+120	0.936
*SUMO2/3*exp (grade dependent) ^#^		0.407	0.398–5.676	0.548
*SUMO2/3* expression *		0.606	0.590–5.694	0.295
*ACT/CS1* (compared to grade 3) *^#^*		−3.941	0.002–0.186	0.001
*Grade 2* (compared to grade 3) *^#^*		−2.064	0.033–0.492	0.003
*SUMO1*exp (grade dependent) ^#^		−0.894	0.112–1.491	0.175
**Recurrence-free survival (10 year)**				
*SUMO1* expression *		0.171	0.552–2.553	0.661
*ACT/CS1* (compared to grade 3) ^#^		−3.063	0.16–0.140	0.000
*Grade 2* (compared to grade 3) ^#^		−1.623	0.083–0.468	0.000
*SUMO2/3*exp (grade dependent) ^#^		−0.897	0.177–0.950	0.038
*SUMO2/3* expression *		−0.026	0.434–2.037	0.875
*ACT/CS1* (compared to grade 3) *^#^*		−3.076	0.015–0.146	0.000
*Grade 2* (compared to grade 3) *^#^*		−1.53	0.092–0.511	0.000
*SUMO1*exp (grade dependent) ^#^		−1.015	0.157–0.939	0.018

## Data Availability

All of the data for this paper can be found in the text and supplementary material.

## Supplementary Materials

The following are available online at https://www.mdpi.com/article/10.3390/cancers13153823/s1, Figure S1: SUMO2/3 expression increases risk for poorer disease outcome in peripheral chondrosarcoma, Figure S3: A heterogeneous group of soft tissue sarcomas show poorer disease outcome with high SUMOylation cascade protein expression, Figure S4: Effect of SUMOylation cascade components and oncogenes (c-MYC, IDH and p53) on sensitivity towards SUMOylation inhibition, Figure S5. Uncropped original western blot, Table S1: Univariate Cox regression analysis results of variates influencing survival.
