# Supplementary material for: SUMOylation Is Associated with Aggressive Behavior in Chondrosarcoma of Bone
Source: Cancers (Basel). 2021 Jul 29;13(15):3823. doi: 10.3390/cancers13153823 (PMC8345166; doi:10.3390/cancers13153823)
Supplement: Supplementary file 1 [file cancers-13-03823-s001.zip › cancers-1313175-supplementary.pdf]

Supplementary Material: SUMOylation is associated with aggressive behavior in chondrosarcoma of bone

Jessie S. Kroonen, Alwine B. Kruisselbrink, Inge H. Briaire-de Bruijn, Olaejirinde O. Olaofe, Judith V. M. G. Bovée and Alfred C. O. Vertegaal

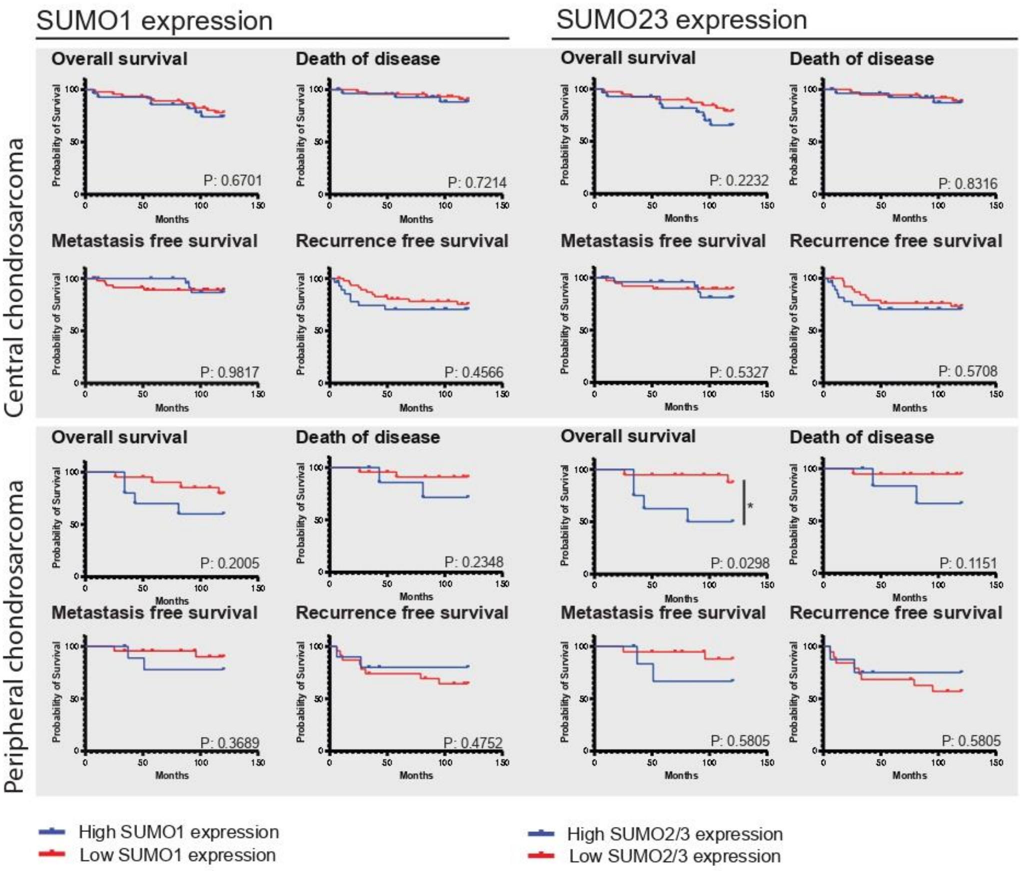

Figure S1: SUMO2/3 expression increases risk for poorer disease outcome in peripheral chondrosarcoma**.** Survival curves showing overall survival, death of disease, metastasis free survival and recurrence free survival separate for central chondrosarcoma and peripheral chondrosarcoma, displaying high expression of SUMO1 or SUMO2/3 versus low expression of SUMO1 or SUMO2/3. SUMO expression was determined based on the scores of the TMAs from Figure 1B**.** Log-rank (Wilson-Cox) analysis was used to calculate significance. *: $p < 0.05$.

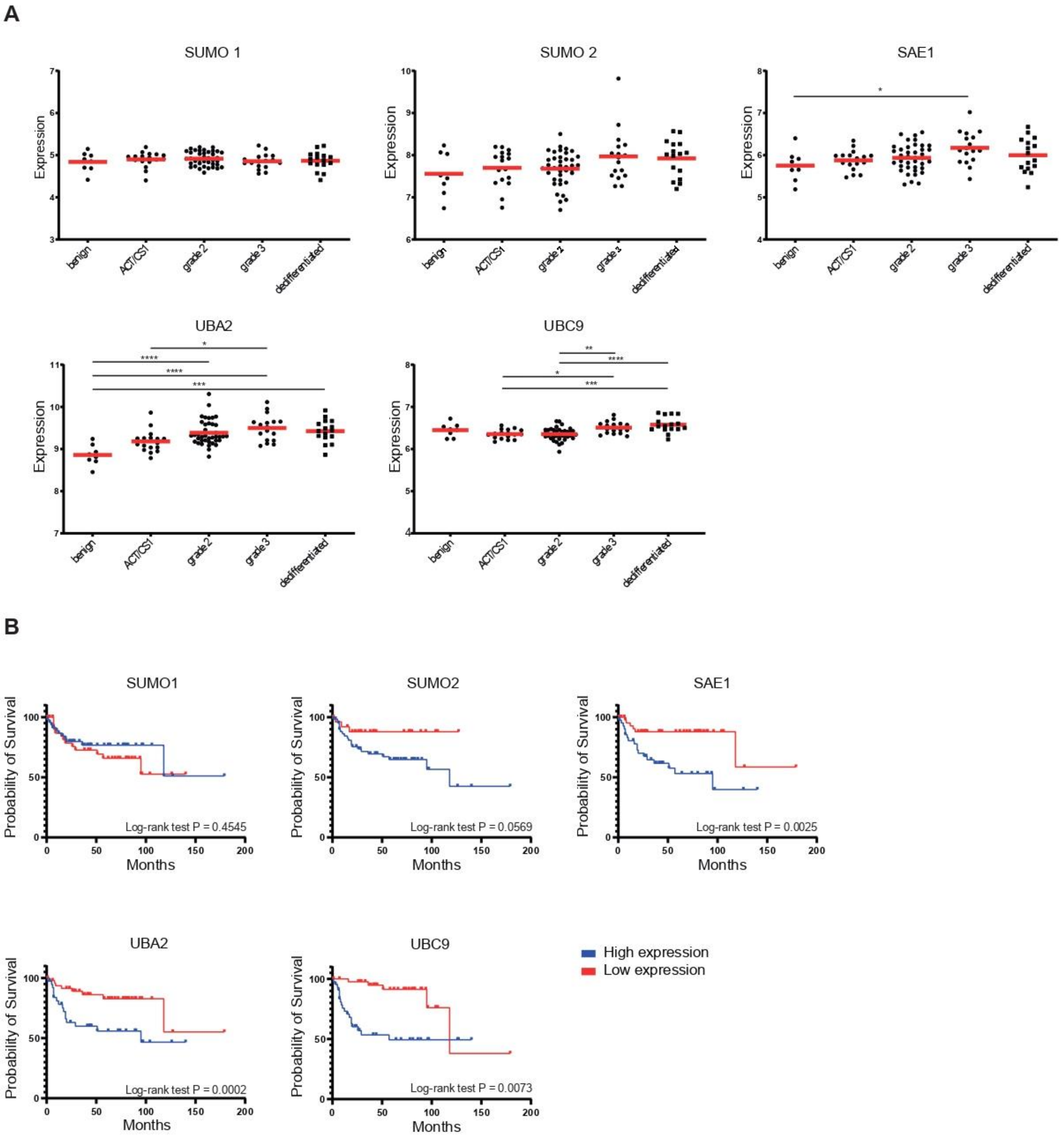

Figure S2. High expression of SUMOylation cascade proteins correlates with tumor aggressiveness and poorer disease outcome in a chondrosarcoma mRNA expression data set. Chondrosarcoma mRNA expression (n=90) from Nicolle et al. 2019 [1] were used to investigate correlations between SUMO pathway (SUMO1-2, SAE1, UBA2 and UBC9) gene expression per histological tumor group (**A**) and survival (**B**). Dot-plots represent mRNA expression presented as mean with standard deviation. * p: < 0.05, **: $p < 0.01$, ***: $p < 0.001$, ****: $p < 0.0001$. Each dot represents mRNA gene expression for one sample. P values were calculated using one-way ANOVA followed by Tukeys multiple comparison. Correlations between mRNA expression and survival were drawn in Kaplan-Meier plots and Log-rank analysis was shown in the graphs. The median per gene was used as cut-off between high and low expression. Log-rank (Wilson-Cox) analysis was used to calculate significance. Cox-regression analysis for the survival data shown in B is depicted in Table S1.

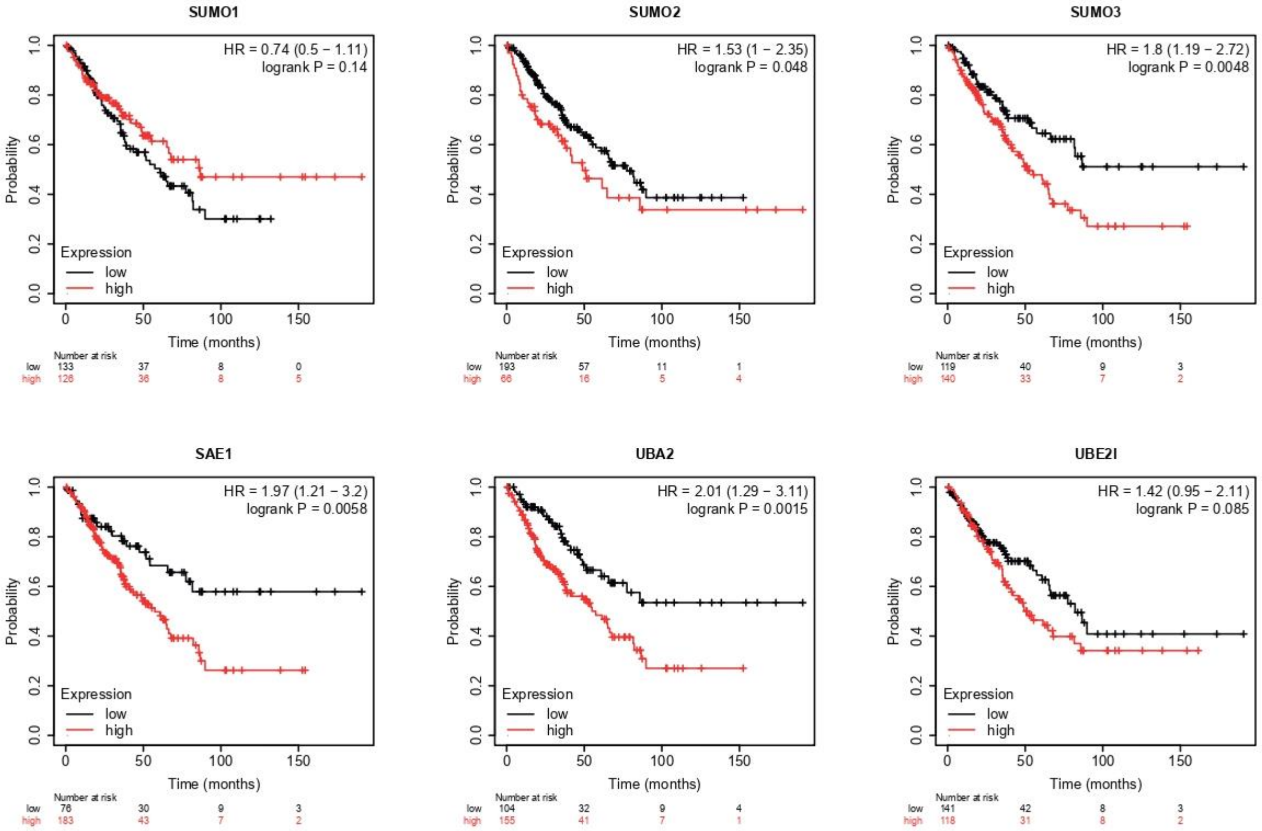

Figure S3: A heterogeneous group of soft tissue sarcomas show poorer disease outcome with high SUMOylation cascade protein expression. Sarcoma RNAseq and survival data (n=259) from the TCGA repository were used to investigate correlations between SUMO pathway (SUMO1-3, SAE1, UBA2 and UBE2) gene expression and survival. Correlations between mRNA expression and survival were calculated using Cox proportional hazard regression and to draw Kaplan-Meier plots. Cut-offs were computed as best-cut offs out of the data set by the kmplot.com algorithm.[2].

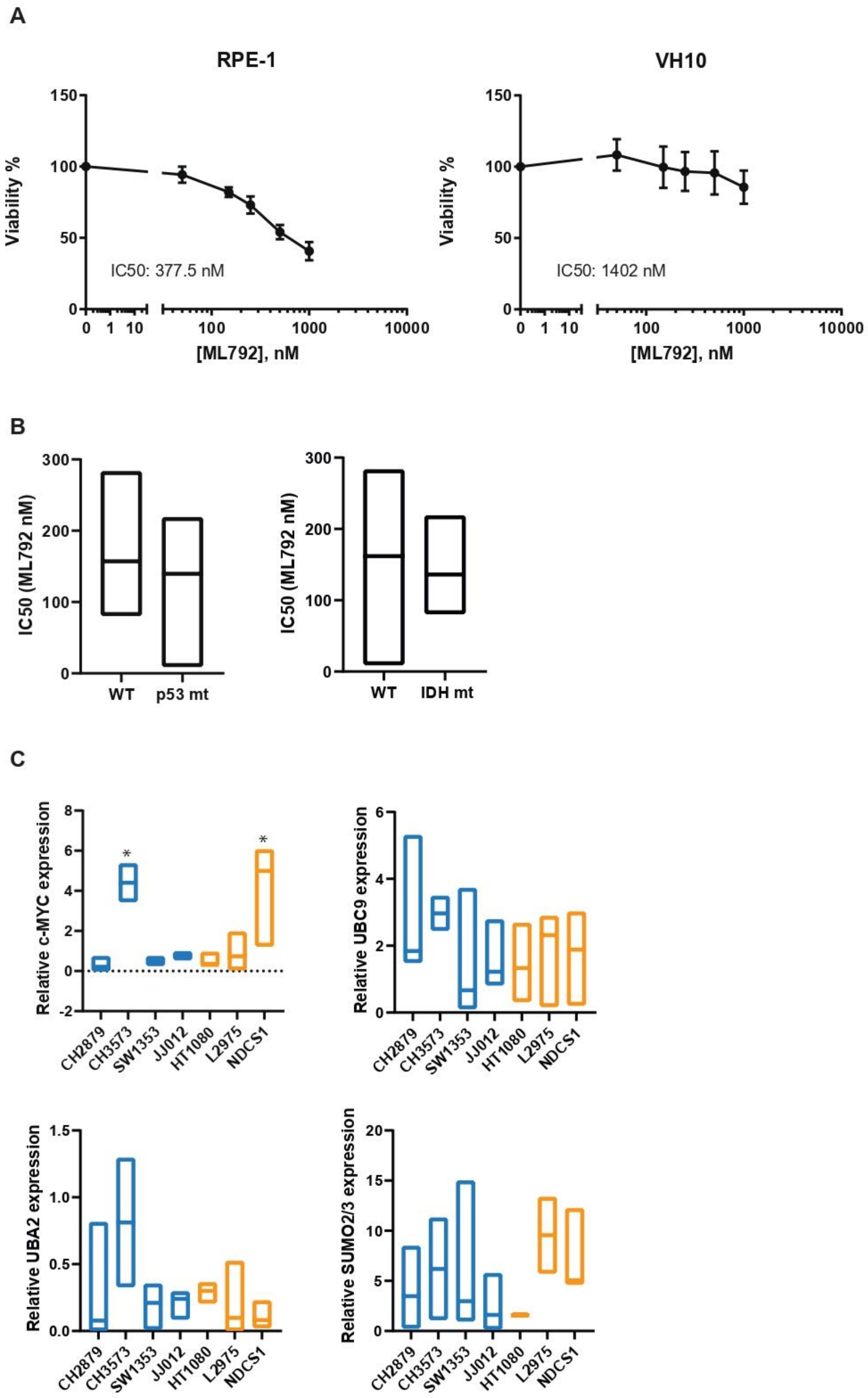

Figure S4. Effect of SUMOylation cascade components and oncogenes (c-MYC, *IDH* and p53) on sensitivity towards SUMOylation inhibition. (**A**) Cell viability assay. RPE-1 and VH10 cells were treated with ML792 and incubated for 3 days. Relative cell viability was studied using Presto-Blue and is represented as mean with standard deviation ($n = 3$). (**B**) Boxplot

to compare ML792 IC50 values of chondrosarcoma cell lines with *IDH* mutations as shown in Figure 3C (n = 4) and wildtype chondrosarcoma (n = 3) cell lines and a boxplot to compare ML792 IC50 values of chondrosarcoma cell lines with P53 mutation as shown in Figure 3C (n = 4) and wildtype chondrosarcoma (n = 3). (**C**) Expression quantification. Relative expression is displayed per protein from C, corrected for γ-tubulin (n = 3). Differences were calculated using one-way ANOVA followed by Tukeys multiple comparison (*: $p < 0.05$).

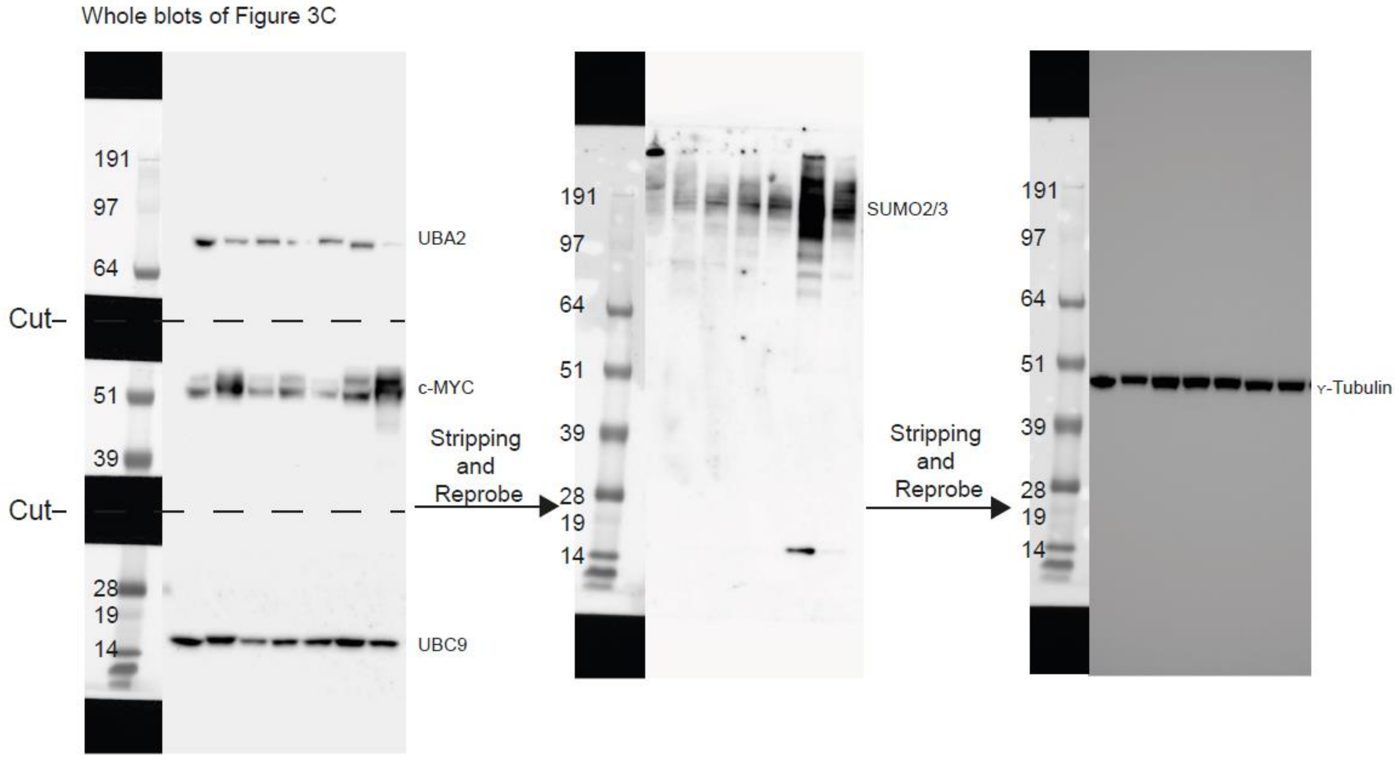

Densitometry readings and intensity ratio to γ-Tubulin

	γ-Tubulin	c-Myc		UBA2		UBC9		SUMO2/3	
Cell line	Intensity	Intensity	Ratio	Intensity	Ratio	Intensity	Ratio	Intensity	Ratio
CH2879	1.042	0.764	0.733205	0.847	0.81286	1.918	1.840691	0.283	0.271593
CH3573	0.731	2.524	3.452804	0.242	0.331053	1.797	2.458276	0.821	1.123119
SW1353	1.159	0.557	0.480587	0.408	0.352028	0.774	0.667817	1.14	0.983607
JJ012	0.94	0.914	0.97234	0.083	0.088298	1.147	1.220213	1.517	1.61383
HT1080	1.002	0.315	0.314371	0.363	0.362275	1.336	1.333333	1.55	1.546906
L2975	0.903	1.767	1.956811	0.471	0.521595	2.092	2.316722	8.636	9.563677
NDCS1	0.723	3.61	4.993084	0.018	0.024896	1.361	1.882434	3.365	4.654219

Figure S5. Uncropped original western blot. Whole blots are provided with intensity readings and intensity ratio to γ-Tubulin from the western blots of Figure 3C.

Table S1. Univariate Cox regression analysis results of variates influencing survival.

Variable	Hazard Ratio (B)	CI (95%) (exp(B))	*p* value
Dichometized dataset			
SUMO1 expression	−0.305	0.330–1.647	0.457
SUMO2 expression	0.556	0.762–3.998	0.118
SAE1 expression	1.289	1.437–9.168	0.006
UBA2 expression	1.107	1.292–7.092	0.011
UBE2I expression	1.681	1.991–14.485	0.001

Continuous representation of mRNA expression			
SUMO1 expression	−1.588	0.028–1.487	0.117
SUMO2 expression	1.204	1.507–7.380	0.003
SAE1 expression	2.011	2.571–21.708	0.000
UBA2 expression	1.672	1.807–15.683	0.002
UBE2I expression	5.124	16.701–1689.159	0.000

References

1. Nicolle, R.; Ayadi, M.; Gomez-Brouchet, A.; Armenoult, L.; Banneau, G.; Elarouci, N.; Tallegas, M.; Decouvelaere, A.-V.; Aubert, S.; Rédini, F.; et al. Integrated molecular characterization of chondrosarcoma reveals critical determinants of disease progression. *Nat. Commun.* **2019**, *10*, 4622, doi:10.1038/s41467-019-12525-7.
2. Nagy, Á.; Munkácsy, G.; Győrffy, B. Pancancer survival analysis of cancer hallmark genes. *Sci. Rep.* **2021**, *11*, 6047, doi:10.1038/s41598-021-84787-5.
